# A new species of *Aspidophryxus* (Isopoda, Dajidae), ectoparasitic on *Mysidella hoshinoi* (Mysidae) in Japan

**DOI:** 10.3897/zookeys.646.10701

**Published:** 2017-01-19

**Authors:** Michitaka Shimomura

**Affiliations:** 1 Kitakyushu Museum of Natural History and Human History, Kitakyushu 805-0071, Japan

**Keywords:** Aspidophryxus, Dajidae, Izu-Oshima Island, Mysidella, parasite

## Abstract

A new dajid, *Aspidophryxus izuensis* **sp. n.**, is described from seven females and six males found infesting the dorsal carapaces of specimens of *Mysidella hoshinoi* Shimomura, 2016 (Mysidae: Mysidellinae) associated with an unidentified species of sea anemone (Haloclavidae) from Izu-Oshima Island, Sagami Sea, central Japan. *Aspidophryxus izuensis* sp. n. differs from its congeners in having a body length about as long as wide, widest at the anterior part in females; an elongate frontal part of the cephalon, half as long as wide in females; the frontal margin of the cephalon exceeding the anterior margins of lateral lamellae in females; an unsegmented, vermiform, elongate pleon in females; and a uropod composed of a protopod and an inner and outer ramus in males. A key to worldwide species in the genus is provided.

## Introduction

Dajidae, a family of the suborder Cymothoida, consists of 54 species belonging to 18 genera, all of which are exclusively ectoparasites of mysid, euphausiid and decapod crustaceans (Boyko and Schotte 2013; Shields and Gómez-Gutiérrez 1996; Williams and Boyko 2012). Dajidae exhibits highest diversity in the Antarctic (seven species) with 14 species in the North East Atlantic regions combined (Williams and Boyko 2012). Seven dajid species have so far been recorded from Japan: *Aspidophryxus japonicus* Shimomura & Ohtsuka, 2011 from the mysid *Holmesiella affinis* Ii, 1937, *Heterophryxus appendiculatus* G. O. Sars, 1885 from the euphausiid *Euphausia recurva* Hansen, 1905, *Holophryxus fusiformis* Shiino, 1937 from the sergestid *Sergestes prehensilis* Bate, 1881, *Notophryxus ocellatus* Shimomura & Ohtsuka, 2011 from the mysid *Rhopalophthalmus orientalis* O. S. Tattersall, 1957, *Prodajus bilobatus* Shiino, 1943 from the mysid *Anisomysis ijimai* Nakazawa, 1910, *Prodajus curviabdominalis* Shimomura, Ohtsuka & Naito, 2005 from the mysid *Siriella okadai* Ii, 1964, and *Zonophryxus retrodens* Richardson, 1903 from the pandalid shrimp *Plesionika semilaevis* Bate, 1888 (Shimomura et al. 2010).

During a parasitological survey of invertebrates in Japanese waters, new parasitic isopods were found on the carapace of the mysid *Mysidella hoshinoi* Shimomura, 2016. The present paper describes a new species of *Aspidophryxus* and is the second occurrence of the genus from Japan.

## Material and methods

Host mysids were collected by a local SCUBA diver using sealable plastic bags (20 cm × 20 cm) by scooping seawater from around a sea anemone (Haloclavidae sp). All specimens obtained were preserved in 80% ethanol. Dajids were removed from hosts under a stereomicroscope. Each individual was dissected and prepared for observation with a light microscope (Nikon E600). For SEM observation (Hitachi S-3000N), specimens were dehydrated through an alcohol series, freeze-dried and sputter-coated with platinum. Total length as indicated in “Material examined” was measured from the tip of the cephalon to the end of the body excluding the pleon. The authors and dates of dajid taxa are referenced but those of the hosts are not. The terminology follows Shimomura et al. (2005).

The type specimens are deposited in the Kitakyushu Museum of Natural History and Human History, Japan (KMNH).

## Systematics

### Aspidophryxus

Taxon classificationAnimaliaIsopodaDajidae

G. O. Sars, 1883

Aspidophryxus G. O. Sars, 1883: 72–73.

#### Type species.

*Aspidophryxus peltatus* G. O. Sars, 1883 (by original designation).

#### Species included.

*Aspidophryxus discoformis* Boyko & Williams, 2012; *Aspidophryxus frontalis* Bonnier, 1900; *Aspidophryxus izuensis* sp. n.; *Aspidophryxus japonicus* Shimomura & Ohtsuka, 2011; *Aspidophryxus peltatus* G. O. Sars, 1883.

### Aspidophryxus izuensis sp. n.

Taxon classificationAnimaliaIsopodaDajidae

http://zoobank.org/406F5790-697C-4411-8E0C-1FB94D4E26E6

Figs 1 , 2 , 3 , 4

#### Material examined.

Holotype. Ovigerous ♀ (1.4 mm) (KMNH IvR 500908), Akino-hama, Izu-Oshima Island, Sagami Sea, Japan, 23 August 2014, 35 m.

Allotype. 1 ♂(KMNH IvR 500911), obtained from the holotype.

Paratypes. 2 ovigerous ♀♀, 1.3 mm (KMNH IvR 500909), 1.2 mm (KMNH IvR 500910), 2 ♂♂, 0.5 mm (KMNH IvR500912), obtained from the female (KMNH IvR 500909), 0.5 mm (KMNH IvR500913), obtained from the female (KMNH IvR 500910), data same as holotype; 2 ovigerous ♀♀, 1.5 mm (KMNH IvR 500914), 1.5 mm (KMNH IvR 500915), 2 ♂♂, 0.5 mm (KMNH IvR500916), obtained from the female (KMNH IvR 500914), 0.5 mm (KMNH IvR500917), obtained from the female (KMNH IvR 500915), Akino-hama, Izu-Oshima Island, Sagami Sea, Japan, 9 August 2014, 35 m; 1 non-ovigerous ♀, 0.8 mm (KMNH IvR 500918), Akino-hama, Izu-Oshima Island, Sagami Sea, Japan, 19 August 2014, 35 m; 1 ovigerous ♀, 0.5 mm (KMNH IvR 500919), 1 ♂, 0.5 mm (KMNH IvR500920), obtained from the female (KMNH IvR 500919), Akino-hama, Izu-Oshima Island, Sagami Sea, Japan, 26 August 2014, 35 m.

#### Diagnosis.

Female: body length and width subequal, anteriorly widest; frontal margin of cephalon exceeding anterior margins of lateral lamellae; frontal part of cephalon half as long as wide; pleon unsegmented, vermiform, elongate half as long as total body length. Male: cephalon fused with first pereomere; uropod composed of protopod and inner and outer ramus.

#### Description of female.

Body (Figs 2A, B, 5A) semicircular, anteriorly widest, 1.1 times as long as maximum width (including lateral lamellae), moderately vaulted dorsally, with pair of broad lateral lamellae filled with 145 eggs; lateral lamellae not reaching beyond frontal margin of cephalon. Egg diameter ranged from 72.8 to 88.4 µm (N = 20; average = 81.8±3.9). Cephalon (Fig. 2B; see Fig. 5A, B) oriented ventrally, without eyes; frontal part long, half as long as wide; anterior margin rounded; posterior margin not visible in dorsal view. Pereon (Fig. 2A) without transverse folds. Medioventral edge of lateral lamellae produced into two digitiform extensions anterior to insertion of pleon (Figs 2B, 5C, E); posterior extension with many scale-like wrinkles. Pleon (Figs 2B, 5A, D–F) segments fused, vermiform, very long, reaching beyond posterior margin of lateral lamellae, with many pits, without lateral plates or pleopods. Pleotelson fused with pleon, without uropods. Antennule and antenna (Figs 3A, 5B) composed of one article each with three setae distally. Oral cone (Figs 3A, 5B) conical, extending beyond surface of cephalon. First to fifth pereopods (Figs 4A, 5B) similar in shape, first pair slightly smaller than others: basis longest; ischium approximately 0.7 times as long as basis; merus fused with carpus; propodus ovate, without setae or spines; dactylus with curved claw.

**Figure 1.**
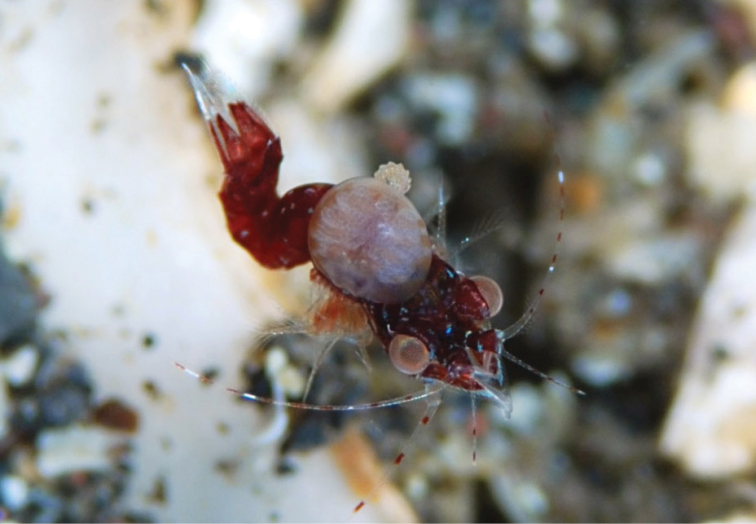
A female and male of *Aspidophryxus izuensis* sp. n. on the carapace of the mysid *Mysidella hoshinoi*, Akino-hama, Izu-Oshima Island, Sagami Sea, Japan, 1 January 2015, 35 m depth, habitus in situ, photographed by O. Hoshino.

**Figure 2.**
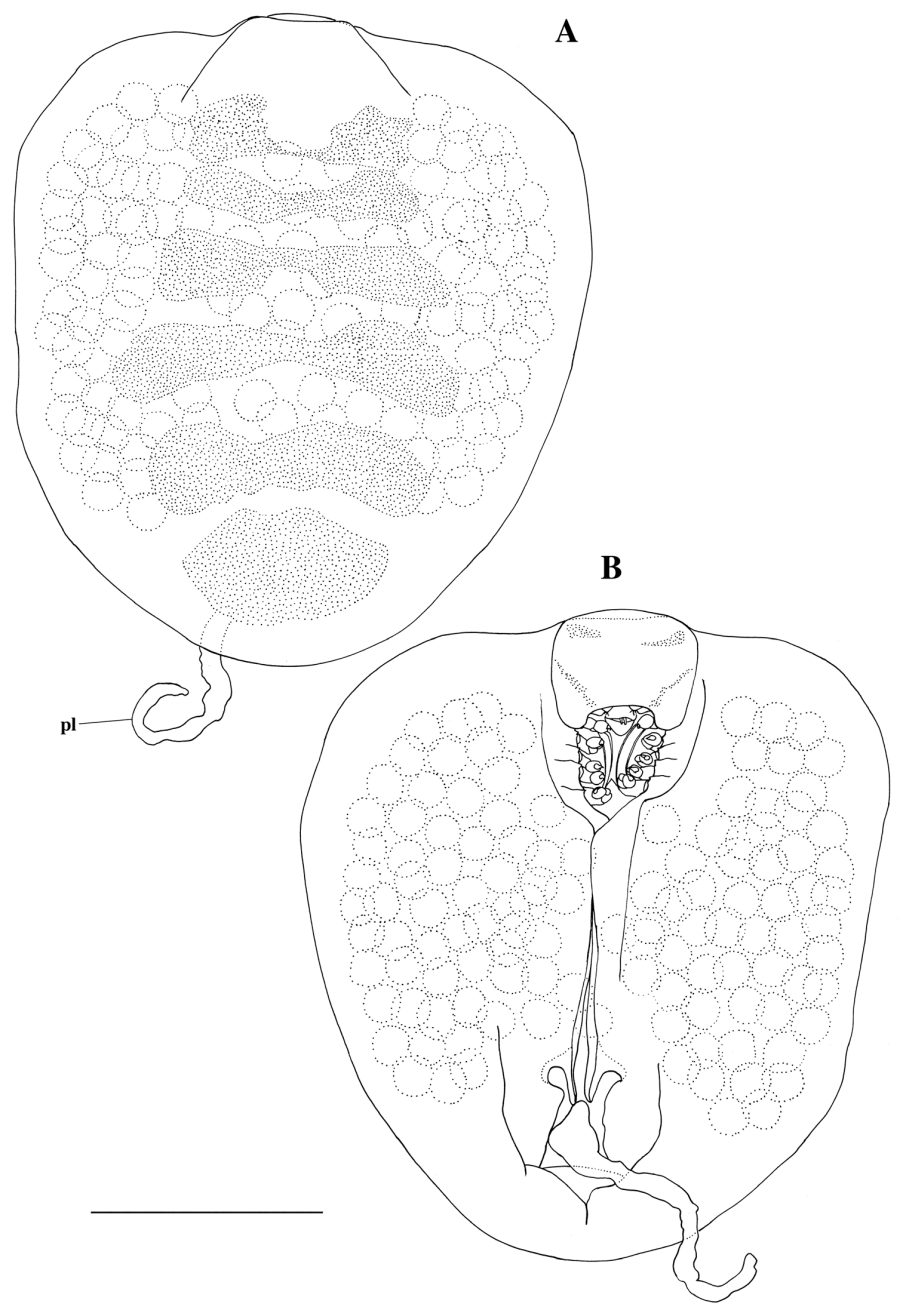
*Aspidophryxus izuensis* sp. n., holotype female: **A** habitus, dorsal **B** habitus, ventral. Scale bar 500 μm. Abbreviation: pl, pleon.

**Figure 3.**
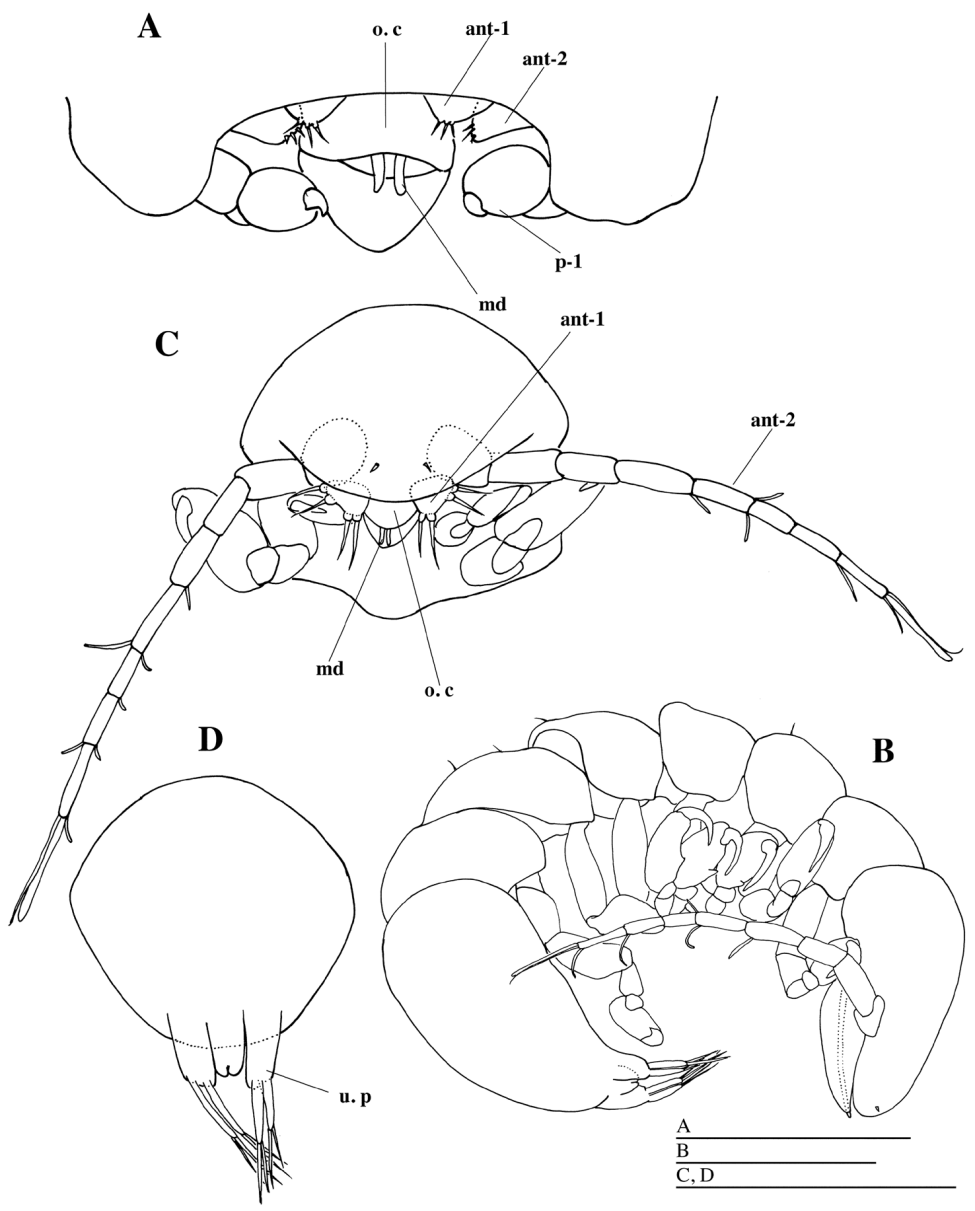
*Aspidophryxus izuensis* sp. n., **A** holotype female **B–D** allotype male (KMNH IvR 500911): **A** cephalon, ventral **B** habitus, lateral **C** cephalon and first and second pereomeres, ventral **D** pleotelson, dorsal. Scale bars 100 μm. Abbreviations: ant-1, antennule; ant-2, antenna; o. c, coral cone; md, mandible; u. p, uropod.

**Figure 4.**
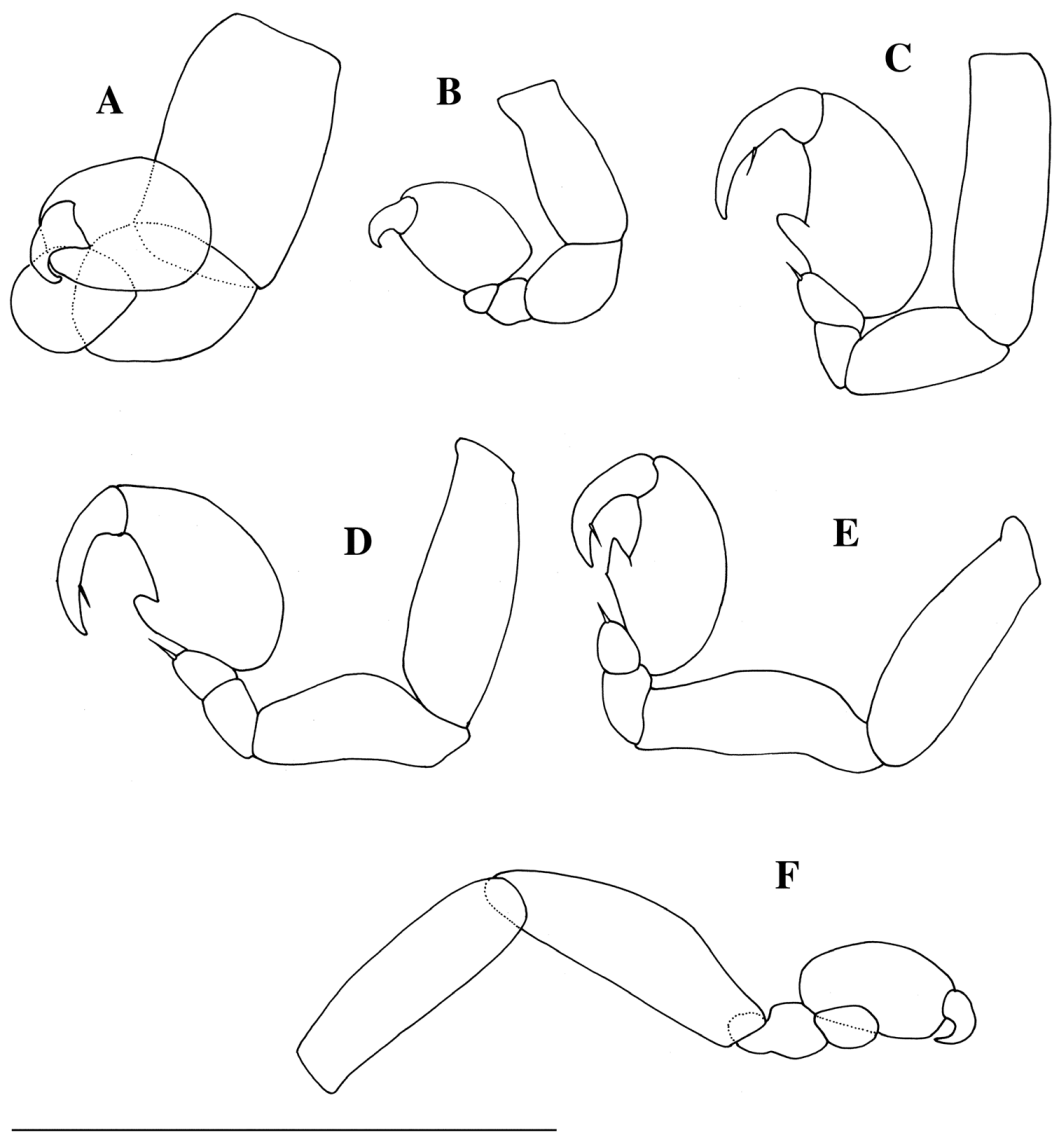
*Aspidophryxus izuensis* sp. n., **A** holotype female **B–F** allotype male (KMNH IvR 500911): **A** left fifth pereopod, dorsal **B** left first pereopod, lateral; **C** left second pereopod, lateral **D** left fifth pereopod, lateral **E** left sixth pereopod, lateral **F** left seventh pereopod, medial. Scale bar 100 μm.

**Figure 5.**
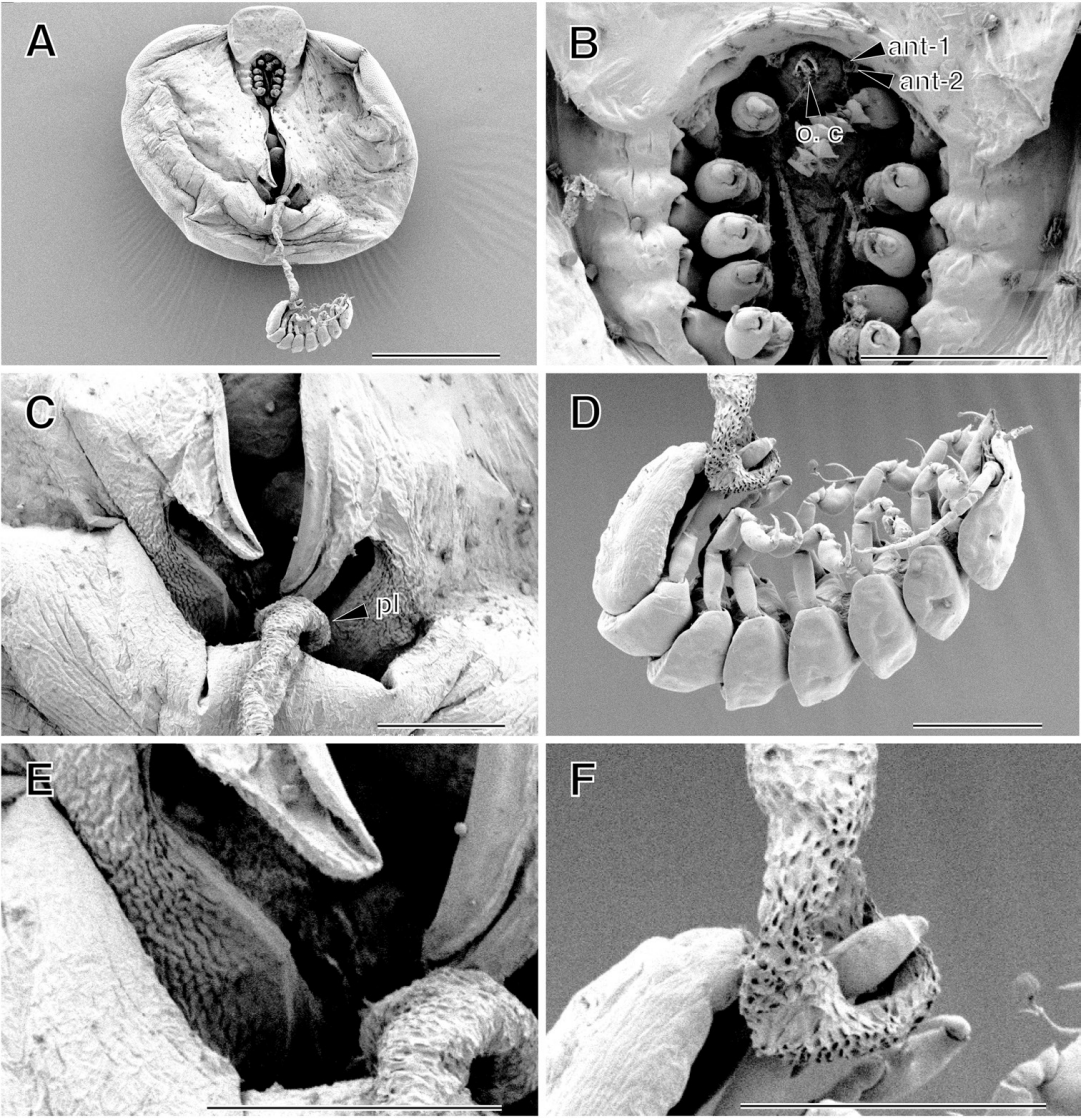
*Aspidophryxus izuensis* sp. n., **A** paratype female (KMNH IvR 500909) and male (KMNH IvR 500912) **B, C, E, F** paratype female (KMNH IvR 500909) **D** paratype male (KMNH IvR 500912). **A** habitus, female, ventral, male, dorsal; **B** cephalon and pereopods, ventral **C** pleon and digitate extensions of oostegites, ventral **D** habitus (clinging to female pleon by seventh pereopods), lateral **E** pleon and digitate extensions of oostegites, ventral; **F** female pleon, ventral. Scale bars **A**, 500 μm; **B–D**, 100 µm. Abbreviations: ant-1, antennule; ant-2, antenna; o. c, oral cone; pl, pleon.

#### Description of male.

Body (Figs 3B, 5D) curved ventrally; scattered setae present dorsally. Cephalon (Fig. 3B, C) without eyes, fused with first pereomere; anterior margin convex. Second to seventh pereomeres (Fig. 3B) separated, subequal in width. Pleon (Fig. 3B, D) unsegmented, slit-like anal cone between uropods. Uropods (Fig. 3B, D) well developed, long, composed of protopod and inner and outer ramus: protopod cylindrical, without setae; inner and outer ramus shorter than protopod, each with two setae distally. Antennule (Fig. 3C) composed of single triangular article, with two distal and two lateral setae. Antenna (Fig. 3C, D) composed of eight articles: first article largest; second article as long as first article; third article shorter than second article; fourth to seventh articles each with one or two setae distally; eighth article with one short and one long setae and one aesthetasc apically. Oral cone (Fig. 3C): pair of mandibular gnathobases protruding from mouth opening. First pereopod (Fig. 4B) smaller than all other pereopods, basis longest; ischium 0.7 times as long as basis; merus trapezoidal; carpus smallest; propodus ovate, without setae; dactylus short, curved inward. Second to fifth pereopods (Fig. 4C, D) similar in shape: basis longest; ischium shorter than basis; merus trapezoidal; carpus smallest, with one seta distally; propodus ovate, with one projection proximoventrally; dactylus long, slightly curved inward, with one seta ventrally. Sixth pereopod (Fig. 4E) propodus slightly smaller than those of second to fifth pereopods. Seventh pereopod (Fig. 4F) longer than all other pereopods: propodus smaller than propodus of sixth pereopod; dactylus short, curved inward, without setae.

#### Color in life.

Female (Figs 1; see Fig. 2A) whitish and translucent, with six transverse translucent light orange bands dorsally. Male (Fig. 1) whitish and translucent.

#### Remarks.

*Aspidophryxus izuensis* sp. n. can be identified by the following combination of characters: body length and width subequal, anteriorly widest in female; frontal margin of the cephalon exceeding anterior margins of lateral lamellae in female; pleon unsegmented, vermiform, very long in female; uropod composed of protopod and inner and outer ramus in male.

*Aspidophryxus izuensis* is most similar to *Aspidophryxus discoformis* Boyko & Williams, 2012, from Caribbean waters (Boyko and Williams 2012) in having the frontal margin of cephalon exceeding the anterior margins of the lateral lamellae and body length and width being subequal. *Aspidophryxus izuensis*, ﻿however, differs from *Aspidophryxus discoformis* by the following characters (those of *Aspidophryxus discoformis* in parentheses): body widest at anterior part in female (widest at middle); pleon very long, reaching beyond posterior margin of lateral lamellae in female (moderately short, not reaching beyond posterior margin of lateral lamellae); frontal part of cephalon long, half as long as wide in female (short, 0.12 times as long as wide); uropods well developed, long, composed of protopod and inner and outer ramus in male (rudimentary, short, uniramous).

Dajid males are found attached to the pleon, lateral plate, or pleopods of the females by the pereopods (Giard and Bonnier 1889; Shimomura et al. 2005). In *Aspidophryxus izuensis*, the males cling to near the end of the pleon of females by seventh pereopods. The pleon of the female has scale-like wrinkles and many pits on its surface; these features might enable males to more easily cling to the surface. This is one of the first reports on how males attach to the pleon of females in the Dajidae.

#### Etymology.

The new species is named after the type locality.

### Key to the females of *Aspidophryxus* species (modified from Boyko and Williams 2012)

**Table:** 

1	Frontal margin of cephalon exceeding anterior margins of lateral lamellae	**2**
–	Frontal margin of cephalon not exceeding anterior margin of lateral lamellae	**3**
2	Body length and width subequal, antenna a single lobe, pleon unsegmented, vermiform	**4**
–	Body longer than wide, antenna segmented, pleon segmented	***Aspidophryxus japonicus***
3	Frontal margin of cephalon rectangular and subequal to mouth/pereopod region in size, body longer than wide	***Aspidophryxus peltatus***
–	Frontal margin of cephalon irregular shaped, much smaller than mouth/pereopod region, body length and width subequal	***Aspidophryxus frontalis***
4	Body widest at middle part, pleon moderately short, not reaching beyond posterior margin of lateral lamellae	***Aspidophryxus discoformis***
–	Body widest at anterior part, pleon elongate, reaching beyond posterior margin of lateral lamellae	***Aspidophryxus izuensis* sp. n.**

## Supplementary Material

XML Treatment for Aspidophryxus

XML Treatment for Aspidophryxus izuensis
